# Intraoperative cell salvage for obstetrics: a prospective randomized controlled clinical trial

**DOI:** 10.1186/s12884-020-03138-w

**Published:** 2020-08-07

**Authors:** Ye Liu, Xiaoguang Li, Xiangming Che, Guosheng Zhao, Mingjun Xu

**Affiliations:** Department of Anaesthesiology, Beijing Obstetrics and Gynecology Hospital, Capital Medical University, 100026 Beijing, China

**Keywords:** Adverse events, Allogeneic blood transfusion, Caesarean section, Intraoperative cell salvage, Postpartum haemorrhage

## Abstract

**Background:**

The latest basic studies and clinical evidence have confirmed the safety and efficacy of intraoperative autologous blood cell transfusion in cardiac surgery and orthopaedics. However, in caesarean section, there are still concerns about the contamination of amniotic fluid and foetal components, and consequently the application of intraoperative autologous blood cell transfusion is not universal. Therefore, this study aimed to evaluate the clinical value of intraoperative autologous blood cell transfusion in obstetric surgery.

**Methods:**

A prospective, randomized, controlled, feasibility study was performed in women undergoing caesarean section. One hundred sixteen participants were randomly assigned at a 1:1 ratio into either the intraoperative cell salvage group or the control group. Allogeneic blood cells were transfused into patients with haemoglobin concentrations < 80 g/dL in both the intraoperative cell salvage group and the control group.

**Results:**

No significant differences were found between the two groups in age, weight, maternal parity, history of previous caesarean section, gestational weeks of delivery, etc. However, compared with the control group, patients in the intraoperative cell salvage group had a significantly lower amount of allogeneic blood cell transfusion, lower incidence of postoperative incision infection, delayed wound healing, perioperative allergy, adverse cardiovascular events, hypoproteinaemia and shorter hospital stay.

**Conclusion:**

The results of this study suggest that the use of autologous blood cell transfusion is safe and effective for patients with obstetric haemorrhage. Trial registration: All procedures performed in studies involving human participants were in accordance with the ethical standards of the Institutional and/or National Research Committee of Beijing Obstetrics and Gynecology Hospital, Capital Medical University (2016-XJS-003-01) as well as the 1964 Helsinki Declaration and its later amendments or other comparable ethical standards. The clinical trials were registered (ChiCTR-ICC-15,007,096) on September 28, 2015.

## Background

Intraoperative cell salvage (ICS) or autologous blood cell transfusion is the practice of recovering red blood cells from blood lost in the operative field and returning them to the patient. It is the most effective form of transfusion that avoids and reduces blood-borne diseases related to transfusion [1]. This technique can significantly reduce allogeneic blood transfusion, which has been widely used in cardiac surgery and orthopaedics. However, given the concerns regarding the contamination of amniotic fluid and foetal components, the application of autologous blood cell transfusion is not universal in caesarean sections [2]. According to studies around the world, amniotic fluid components can be almost completely removed through the washing process of autologous blood recovery and filtration using leukocyte-removing filters. Recently, intraoperative cell salvage infusion has been gradually applied overseas in patients with a broader range of obstetric diseases, including placenta previa, multiple caesarean sections, previous history of obstetric haemorrhage, rejection of allogeneic transfusion and severe preoperative anaemia [3, 4].

Although in China, the application of recycled auto-transfusion in obstetrics is taboo, most domestic scholars agree that when intractable massive haemorrhage occurs during caesarean section or it is difficult to implement allogeneic transfusion, intraoperative auto-transfusion using an autologous-blood-recycling machine combined with leukocyte-removing filters should be recommended. Therefore, it is essential to assess the safety and effectiveness of recycled auto-transfusion in obstetrics.

## Methods

**Fig. 1:**
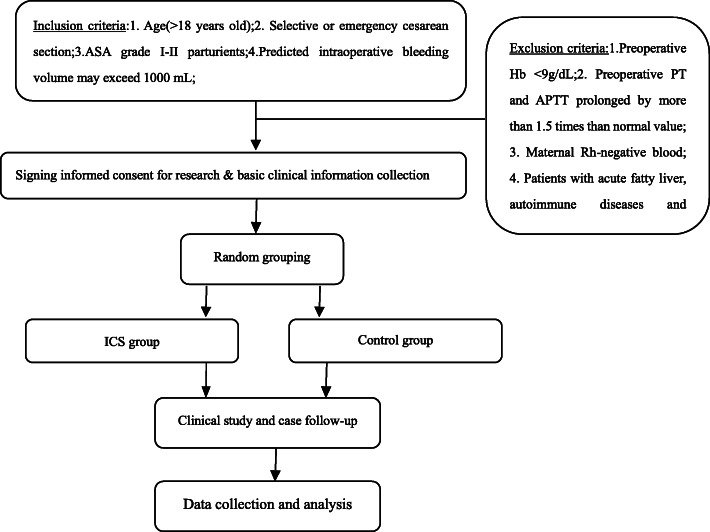
Flow diagram of the study

A prospective, randomized, controlled study was performed to evaluate the feasibility of intraoperative auto-transfusion using an autologous-blood-recycling machine (BW-8200B, Wandongkangyuan, China) combined with a leukocyte-removing filter (Nanjing Shuangwei, China) filter for women undergoing caesarean section between July 2016 and May 2019. A total of 116 participants were randomly assigned at a 1:1 ratio into either the intraoperative cell salvage (ICS) group or the control group. Figure 1 shows the schematic diagram of the trial. Preeclampsia was defined as a unique disorder of gestation and multiple systems associated with systolic blood pressure > 140 mmHg and diastolic blood pressure > 90 mmHg as well as proteinuria > 1 (0.3 g per 24 h) and gestational age > 37 weeks. Placenta previa was defined as a condition where the placenta lies low in the uterus and partially or completely covers the cervix. Abnormally invasive placenta (AIP) was defined as a placenta that does not separate spontaneously at delivery.

Patient identification: After informed consent was obtained, a dedicated patient identification number (PID) was assigned to each patient for patient identification throughout the study process. According to the random number table generated by a computer, the research centre then performed random grouping. Then patients were grouped according to their assigned number. This clinical trial was approved by the Hospital Ethical Review Committee (No. 2016-XJS-003-01) and was registered (ChiCTR-ICC-15,007,096) on September 28, 2015. Details of the study can be found at http://www.chictr.org.cn/showproj.aspx?proj=11283.

### Inclusion and exclusion criteria

Puerpera were enrolled in the study if they (1) were > 18 years old and diagnosed as American Society of Anaesthesiologists (ASA) Classification I-II; (2) had undergone elective or emergency caesarean section; and (3) had estimated intraoperative bleeding > 1000 mL. Puerpera were excluded if they (1) had preoperative Hb < 10 g/dL, preoperative platelets < 50 × 10^9^/L, > 1.5 times prolonged PT and APTT before surgery, or body weight < 50 kg, (2) were Rh-negative or complicated with haematological diseases, autoimmune diseases, or acute fatty liver/HELLP syndrome; (3) had undergone preoperative anticoagulation therapy and accepted allogeneic blood transfusion within 3 months before the study, and participated in other clinical studies during pregnancy; (4) refused to accept allogeneic blood transfusion; and (5) had any diseases that could lead to inability to cooperate with the study, such as mental illness, language-comprehension disorders, etc.

### Expected sample size

The expected sample size was calculated by referring to the studies on the effect of recovery autologous blood transfusion on the prognosis of orthopaedics and cardiac surgery patients with the smallest difference in haemoglobin index before and after selection as the basis for measuring the sample size, and setting β = 0.1 and ɑ=0.05 in the one-sided test method, level = 0.05 and a degree of power of 90%. The effective sample size calculated was 52 cases per group. Considering the drop rate of 10%, the final sample size was 58 cases per group.

### Interventions

Allogeneic red blood cells or recovered autologous blood-washed red blood cells after filtration were transfused to patients with haemoglobin < 80 g/L in the control or ICS groups, respectively. In addition, transfused blood was supplemented with crystal or colloidal fluid for patients with bleeding blood volume < 500 ml.

For patients in the ICS group, an autologous blood recycling machine was installed before the operation. After delivery and placental separation, anticoagulant composed of 25,000 IU of heparin per 1000 ml of 0.9% NaCl solution was drip fed (1 drop/s) into the operation field and allowed to mix with the maternal blood spilt at the time of surgery into the operation field. The blood-saline solution (usually 800 mL) was then recovered using a separate special suction tube of an isolation suction system at a vacuum pressure of 20 KPa into a sterile reservoir [5] and centrifuged to allow larger, dense red blood cells (RBCs) to cling to the wall of the tube, while all other blood components were discarded directly to the waste bag. The RBCs were washed with and resuspended in sterile isotonic sodium chloride (0.9% NaCl) using a blood recycling machine and then infused back into the patient after passing through a white blood cell filter as soon as possible both during and after surgery. Autologous blood was required not to be stored for more than 6 h. During the operation, another suction unit was used to remove amniotic fluid. The technique had been previously standardized in our hospital. Infusion was stopped when the patient’s haemoglobin concentration reached 80 g/L. If the patient’s haemoglobin concentration was still < 80 g/dL, allogeneic red blood cells were infused until the haemoglobin concentration reached 80 g/L.

For patients in the control group, allogeneic red blood cells were infused when the haemoglobin concentration was < 80 g/L. When the patient’s haemoglobin concentration was ≥ 80 g/L, no blood cell transfusion was given. The amount of red blood cell transfusion depended on the bleeding amount and rate as well as the haemoglobin level.

### Indications for the transfusion of other blood products

Intraoperative massive bleeding and massive autologous red blood cell transfusion are often accompanied by the loss of platelets and coagulation factors. In this case, other blood products, such as platelets, fresh frozen plasma and cryoprecipitate, should be appropriately infused. It is recommended that when the amount of bleeding exceeds 3500 mL, fresh frozen plasma (FFP) should be infused. If ordinary frozen plasma is infused, cryoprecipitate should be supplemented appropriately. If the amount of bleeding exceeds 2000 mL, platelet transfusion should be given. Patients were released from the operating room if their haemoglobin concentration was maintained at ≥ 80 g/L.

### Anaesthesia and surgery

General or spinal anaesthesia was applied to all participants of the study by the responsible anaesthesiologist. The type, dose and management of anaesthetics, surgical procedure and ICU treatment were determined by the centre.

### Blood transfusion management and data collection

In addition to intraoperative grouping, different blood transfusion methods were used after the operation. If the haemoglobin concentration was < 80 g/L, allogeneic blood was transfused until the patient’s haemoglobin concentration was ≥ 80 g/L. Perioperative treatments using other blood transfusions were also conducted following the existing guidelines. Blood loss during the operation was calculated by the research team to minimize variation.

### Baseline visits

Patients participating in the study met all inclusion criteria and exclusion criteria and signed the informed consent form. The patient’s important underlying conditions, such as age, body weight, nulliparity, demographic data, weeks of gestation, comorbidities, caesarean section, placental position, intraoperative blood loss, eligibility criteria, and informed consent, were required to be included in the case report form (CRF). Haemoglobin, coagulation function and blood gas analyses were performed before surgery.

### During surgery

During the surgery, vital signs, haemoglobin concentration, volume of allogeneic red blood cell transfusion, autologous blood recovery and transfusion, other blood product transfusion, fluid infusion, urine, type and dose of anaesthetics, and other drug dosages were monitored.

### Clinical outcomes

The following clinical outcomes were recorded: (1) haemoglobin concentration at 30 min, 24 h, 3 d, and 7 d after surgery or at discharge (whichever was earlier); (2) coagulation function (PT/APTT) at 24 h, 3 d, and 7 d after surgery or at discharge (whichever was earlier); (3) results of blood gas analysis at 30 min and 24 h after surgery; (4) blood type antibody screened at 5 d after surgery; (5) in the case that the participants were transferred to the ICU, the cause for the transfer, ICU stay time and cost, use of mechanical ventilation and time; (6) use of allogeneic blood products during hospitalization; and (7) occurrence of amniotic fluid embolism (AFE), sepsis (Sepsis), acute respiratory distress syndrome (ARDS), disseminated intravascular coagulation (DIC), pulmonary embolism (PE), complications of various organ systems and/or complications related to blood transfusion during the postoperative hospitalization; (8) hospitalization time and expenses; (9) complications during follow-up, and (10) completion of the clinical study, death (cause, time), withdrawal (cause) and rejection (cause).

### Statistical analyses

Statistical analyses were performed using SPSS version 17.0 (SPSS Inc., Chicago, IL, USA). The data distribution was tested for normality by visual inspection of histograms and the Kolmogorov-Smirnov test. Continuous variables are described using the mean ± standard deviation (SD). Normally distributed data were compared by the t-test or chi-square test according to the data type, and nonnormally distributed data were tested by nonparametric test. Time events were analysed by Kaplan-Meier curves. All statistical analyses were performed by bilateral tests. A *P* < 0.05 value was considered statistically significant.

## Results

As shown in Table 1, there were no significant differences between the two groups in age, weight, maternal parity, history of previous caesarean section, gestational weeks of delivery, number of placenta previa cases, number of placental penetration cases, number of preeclampsia cases, estimated preoperative blood loss, and number of cases with preoperative abdominal arterial balloon placement.

**Table 1:** Characteristics of the patients

Variable	ICS group (*n* = 58)	Control group (*n* = 58)	*P*
Age, y	35.32 ± 4.61	36.11 ± 4.83	0.815
Weight, kg	67.61 ± 5.38	68.12 ± 5.85	0.687
Nulliparous	10 (17.24%)	9 (15.52%)	0.832
Previous caesarean delivery information	48 (82.76%)	49 (84.48%)	0.940
Pregnancy duration at delivery, weeks	37.12 ± 0.36	36.91 ± 0.33	0.998
Placenta previa	20 (34.48%)	19 (32.76%)	0.890
Abnormally invasive placenta	38 (65.52%)	39 (67.24%)	0.930
Preeclampsia	7 (12.07%)	8 (13.79%)	0.808
Abdominal aorta balloon placement	20 (34.48%)	21 (36.21%)	0.893
Estimated blood loss ≥ 3000 mL	30 (51.72%)	30 (51.72%)	1
Estimated blood loss ≥ 5000 mL	15 (25.86%)	14 (24.14%)	0.868

Data are presented as the mean ± standard deviation (SD) or n (%)

Table 2 shows the comparison of intraoperative conditions between the two groups. There were no significant differences between the two groups in operation time (*P* = 0.937), mean intraoperative bleeding volume (*P* = 0.986), perioperative haemoglobin level (*P* = 0.998), intraoperative crystal and colloidal fluid infusion (*P* = 0.981), or fibrinogen and platelet transfusion (*P =* 0.893, *P* = 0.714). However, compared to the control group, patients in the ICS group had a significantly lower amount of allogeneic blood cell transfusion (*P* = 3.6e^− 5^).

**Table 2:** Comparison of the intraoperative conditions between the two groups

Variable	ICS group (n = 58)	Control group (n = 58)	*P*
Operation time, min	113.52 ± 18.33	120.26 ± 27.55	0.937
Blood loss, ml	2350.68 ± 600.52	2520.12 ± 610.77	0.986
Preoperative haemoglobin	116.52 ± 41.35	115.47 ± 41.11	0.998
Intraoperative haemoglobin minimum	66.85 ± 12.58	64.55 ± 15.63	0.807
Postoperative haemoglobin	100.71 ± 37.33	96.15 ± 33.26	0.943
IOCS, mL	735.67 ± 37.58	N/A	N/A
ABT, mL	820.76 ± 185.93	1370.53 ± 200.65	3.6e^− 5^
Volume of crystalloid infusion, ml	3800.69 ± 150.33	3850.57 ± 180.51	0.981
Volume of colloid infusion, ml	500.38 ± 50.52	500.26 ± 60.73	1
Fibrinogen transfusion	21(36.21%)	20(34.48%)	0.893
Platelet transfusion	4(6.9%)	3(5.17%)	0.714

Data are presented as the mean ± standard deviation (SD) or n (%). *IOCS *Intraoperative cell salvage, *ABT *Allogeneic blood transfusion, *N/A *Non-applicable

Table 3 shows that there were no significant differences in the perioperative coagulation functions between the two groups (*P* = 0.982, *P* = 01, *P* = 01, *P* = 01, *P* = 0.775, *P* = 0.994, *P* = 0.767, *P* = 0.664, *P* = 0.982, *P* = 0.948, respectively).

**Table 3:** Comparison of perioperative coagulation function changes between the two groups

Variable	ICS group (*n* = 58)	Control group (*n* = 58)	*P*
Pre- PT, s	11.78 ± 0.65	11.53 ± 0.61	0.982
Post-PT, s	12.54 ± 0.39	12.17 ± 0.41	1
Pre-APTT, s	29.92 ± 1.38	28.87 ± 1.33	1
Post-APTT, s	31.07 ± 1.56	30.79 ± 1.73	1
Pre-INR	1.26 ± 0.11	1.31 ± 0.13	0.775
Post-INR	1.41 ± 0.18	1.33 ± 0.15	0.994
Pre-Fib, mg/L	3.36 ± 0.32	3.41 ± 0.41	0.767
Post-Fib, mg/L	2.86 ± 0.52	2.91 ± 0.73	0.664
Pre-Plt, 10^9^/L	166.65 ± 15.56	172.63 ± 14.73	0.982
Post-Plt, 10^9^/L	158.87 ± 16.67	163.46 ± 13.26	0.948

Data are presented as the mean ± standard deviation (SD) or n (%). *PT *Prothrombin time, *APTT *Activated partial thromboplastin time, *INR *International normalized ratio, *Fib *Fibrinogen, *Plt *Platelet count, *Pre *Preoperative value, *Post *Postoperative value

As shown in Table 4, compared to the control group, patients in the ICS group had a significantly decreased incidence of postoperative incision infection (*P* = 0.041), delayed wound healing (*P* = 0.041), perioperative allergy, adverse cardiovascular events (*P* = 0.026), and hypoproteinaemia (*P* = 0.015) as well as a shorter hospital stay (*P* = 0.046).

**Table 4:** Comparison of postoperative complications between the two groups

Variable	ICS group (*n* = 58)	Control group (*n* = 58)	P
Hospitalization time, days	5.16 ± 0.23	8.89 ± 1.11	0.046
Wound infection	1 (1.72%)	7 (12.07%)	0.041
Delayed wound healing	1 (1.72%)	7 (12.07%)	0.041
Allergic reaction	2 (3.45%)	10 (17.24%)	0.028
Perioperative adverse cardiovascular events	2 (3.45%)	9 (15.52%)	0.026
Hypoproteinaemia	1 (1.72%)	9 (15.52%)	0.015
Amniotic fluid embolism	0	0	0

Data are presented as the mean ± standard deviation (SD) or n (%)

## Discussion

In China, perinatal maternal death is mainly due to an intraoperative obstetric haemorrhage of 2000 ml or more caused by placenta previa or an abnormally invasive placenta [6–8]. To reduce the incidence of perinatal maternal death, blood transfusion has been widely performed [4]. However, this treatment often results in a significantly increased incidence of complications such as blood transfusion-related allergic reactions and fever, which severely endanger maternal life and increase the occupational risks of obstetric anaesthesiologists [9, 10].

In the event of a massive haemorrhage in non-obstetric surgery, ICS reduces the demand for allogeneic (donor) red blood cells and can be lifesaving if the volume of blood involved has outstripped the local supply [10, 11]. Therefore, ICS has become a common practice in many surgical specialties. However, the safety of ICS in obstetric surgery has yet to be determined [11]. Numerous publications have shown that using a leukocyte-removing filter for filtration during the return process could almost completely eliminate the amniotic fluid component from the recovered blood [12]. At present, some medical institutions around the world have carried out autologous blood recovery from amniotic fluid in the operation field after delivery and placenta separation and filtration of white blood cells without leading to obvious puerperal complications [13]. Although the technique has been confirmed to be safe by multiple studies with large clinical sample sizes [14], its promotion has currently encountered some taboos. For example, pregnant patients with malignant tumours and patients with sickle cell anaemia were excluded. Patients with blood contaminated with bacteria and red blood cells destroyed in large quantities were also excluded.

In our study, the average age of the two groups of women was approximately 35 years, which was higher than the maternal age of other studies due to the liberalization of the two-child policy. Thus, the increase in the maternal age and maternal history of caesarean section has led to an increase in complications such as clinical placenta previa. The gestational weeks of delivery in the two groups were approximately 37 weeks, lower than the gestational weeks of normal delivery. In this study, the proportion of maternal placenta and placenta penetration was as high as 34% and 65%, respectively.

In recent years, with the continuous development of vascular mediator medicine, some scholars have begun to use mediator technology to treat dangerous placenta previa with placenta implantation [15]. After repeated exploration, it was found that the balloon catheter could be placed in the distal abdominal aorta, bilateral common iliac artery or bilateral internal iliac artery of the patient prior to caesarean section. The balloon could be dilated during the operation, which could temporarily block uterine blood flow, therefore reducing bleeding during the operation [16]. This could provide the surgeon with a clear operative field of vision and sufficient time. Suture and ligation were also used to stop bleeding. The balloon should be placed below the renal artery and around the L4 vertebral body to effectively block the blood supply in the pelvic cavity and ensure the blood supply of other tissues and organs [17]. Although blocking the bilateral common iliac artery or internal iliac artery can effectively block the blood supply in the pelvic cavity, balloon implantation of the lower limbs is also needed due to the characterization of greater trauma, longer operation time and larger foetal radiation. Blocking the distal abdominal aorta balloon is simple and can be achieved by unilateral femoral artery puncture [18]. In this study, there were 20 cases of abdominal aortic balloon placement in the ICS group and 21 cases in the control group, showing no significant difference between the two groups (*P* = 0.893).

Goal-directed fluid therapy (GDFT) is an individualized rehydration scheme based on the patient’s sex, age, body mass, disease type, preoperative general condition and volume status [19]. It is an important part of optimal fluid management for high-risk surgical patients. In this study, a high radiocolloid infusion scheme was chosen to minimize colloid infusion and avoid coagulation problems caused by excessive colloid infusion [20]. Our experience is in performing target-oriented liquid therapy according to laboratory indicators as soon as possible to administer sufficient red blood cells, plasma, fibrinogen and other substances [21]. There were no significant differences between the two groups in operation time, levels of preoperative, intraoperative and postoperative haemoglobin or the transfusion rate of allogeneic red blood cells (*P* = 0.937, *P* = 0.998, *P* = 0.807, *P* = 0.943, respectively).

At present, the concentration and washing volume of anticoagulant (heparin) used in intraoperative cell salvage during obstetric surgery are not standardized and inconsistent among the literature [22, 23]. The concentration of anticoagulant (heparin) in intraoperative cell salvage during non-obstetric surgery is 30 IU/ml, and the washing volume is 4–5 times that of the centrifugal tube [24]. However, maternal blood is in a hypercoagulable state in the later period of pregnancy, and the blood collected in the blood storage tank is agglutinated and even blocked. In this study, 50 IU/ml heparin was used to achieve very good clinical results, and there was no significant difference in perioperative coagulation function between the two groups. The results confirmed that recycled blood autotransfusion had no significant effect on the coagulation function of patients. Our study also showed that the incidences of incision infection, delayed wound healing, perioperative allergies, adverse cardiovascular events and hypoproteinaemia were significantly lower, while the length of hospitalization was significantly shorter in the ICS group than in the control group, confirming that a large amount of allogeneic blood cell transfusion would interfere with the immune function of patients and lead to postoperative complications.

The UK SHOT haemovigilance scheme has repeatedly highlighted suboptimal practices in relation to the management of anti-D prophylaxis in cases of caesarean section with rhesus incompatibility. It has stressed the need for improved awareness of national guidelines, supported by education and training among all health-care professionals involved. Therefore, these patients were not included in our study. It is worth noting that filters can cause acute haemodynamic reactions such as transient hypotension [8]. An increase in the rate of AFE has long been considered a potential risk of returning cell-salvaged blood during caesarean sections. It is worth mentioning that no patient in either group had amniotic fluid embolism(AFE). Our study is a prospectively registered, clinical randomized controlled trial that has been robustly conducted, independently monitored, rigorously analysed and transparently reported. In the absence of adverse events, the implementation of a cell saver reduces the need to use precious banked blood. However, it is arguably unethical to carry out a study where a patient is denied autologous cell transfusion in favour of banked donor blood. A criticism of the sample size and power with presumption of the likelihood of a type II error could risk erroneous conclusions.

In summary, the latest domestic and international studies and clinical evidence have shown that intraoperative cell salvage can reduce maternal allogeneic blood transfusion and related complications and have confirmed the effectiveness and safety of intraoperative cell salvage in obstetrics. Because our study is a single-centre study with a small sample size, the safety of intraoperative autologous blood transfusion in obstetrics requires large-scale investigation and needs to be further confirmed by more internationally recognized randomized controlled studies.

## Conclusions

The current research evidence shows that autologous blood transfusion is safer and more effective for patients undergoing caesarean surgery. If new, cheaper or more efficient cell salvage technology becomes available, the conclusions of this study may need to be revisited. The same is true if donor blood shortages should become extreme and acute. However, the findings of this study do not alter the recommendations on the routine use of cell salvage during caesarean section. The future of the use of cell salvage in obstetrics now relies on the clinicians who must decide whether to implement it during elective caesarean deliveries, thereby preparing the team for emergency situations when immediate blood transfusion could save a woman’s life.

## Data Availability

The datasets analysed are available from the corresponding author upon reasonable request.

## Abbreviations

AFE: Amniotic fluid embolism
AIP: Abnormally invasive placenta
ARDS: Acute respiratory distress syndrome
ASA: American Society of Anesthesiologists
CRF: Case report form
DIC: Disseminated intravascular coagulation
FFP: Fresh frozen plasma
GDFT: Goal-directed fluid therapy
ICS: Intraoperative cell salvage
PE: Pulmonary embolism
PID: Patient identification
RBCs: Red blood cells
